# Hyperornithinemia–Hyperammonemia–Homocitrullinuria Syndrome in Vietnamese Patients

**DOI:** 10.3390/medicina60111877

**Published:** 2024-11-16

**Authors:** Khanh Ngoc Nguyen, Van Khanh Tran, Ngoc Lan Nguyen, Thi Bich Ngoc Can, Thi Kim Giang Dang, Thu Ha Nguyen, Thi Thanh Mai Do, Le Thi Phuong, Thinh Huy Tran, Thanh Van Ta, Nguyen Huu Tu, Chi Dung Vu

**Affiliations:** 1 Center of Endocrinology, Metabolism, Genetic/Genomics and Molecular Therapy, Vietnam National Children’s Hospital, 18/879 La Thanh, Dong Da, Hanoi 11512, Vietnam; khanhnn@nch.gov.vn (K.N.N.); ngocctb@nch.gov.vn (T.B.N.C.); giangdk@nch.gov.vn (T.K.G.D.); thuha@nch.gov.vn (T.H.N.); maidtt@nch.gov.vn (T.T.M.D.); 2 Department of Paediatrics, Hanoi Medical University, 1st Ton That Tung Street, Hanoi 11521, Vietnam; 3 Center for Gene and Protein Research, Hanoi Medical University, 1st Ton That Tung Street, Hanoi 11521, Vietnam; tranvankhanh@hmu.edu.vn (V.K.T.); nguyenngoclan@hmu.edu.vn (N.L.N.); phuongle@hmu.edu.vn (L.T.P.); 4 Biochemistry Department, Hanoi Medical University, 1st Ton That Tung Street, Hanoi 11521, Vietnam; tranhuythinh@hmu.edu.vn (T.H.T.); tathanhvan@hmu.edu.vn (T.V.T.); 5 Hanoi Medical Univerity Hospital, Hanoi Medical University, 1st Ton That Tung Street, Hanoi 11521, Vietnam; nguyenhuutu@hmu.edu.vn

**Keywords:** HHH syndrome, *SLC25A15* variant, Vietnamese patients, p.Arg179*, p.Phe188del, p.Met137Cysfs*10

## Abstract

*Background and Objectives*: Hyperornithinemia–hyperammonemia–homocitrullinuria syndrome (HHH; OMIM 238970) is one of the rare urea cycle disorders. Ornithine carrier 1 deficiency causes HHH syndrome, characterized by failure of mitochondrial ornithine uptake, hyperammonemia, and accumulation of ornithine and lysine in the cytoplasm. The initial presentation and time of diagnosis in HHH highly varies. Genetic analysis is critical for diagnosis. *Materials and Methods*: This study encompassed retrospective and prospective analyses of four unrelated Vietnamese children diagnosed with HHH syndrome. *Results*: The age of diagnosis ranged from 10 days to 46 months. All four cases demonstrated hyperornithinemia and prolonged prothrombin time. Three out of four cases presented with hyperammonemia, elevated transaminases, and uraciluria. No homocitrulline was detected in the urine. Only one case depicted oroticaciduria. Genetic analyses revealed three pathogenic variants in the *SLC25A15* gene, with the c.535C>T (p.Arg179*) variant common in Vietnamese patients. The c.562_564del (p.Phe188del) and c.408del (p.Met137Cysfs*10) variants were detected in one case. The latter variant has yet to be reported in the literature on HHH patients. After intervention with a protein-restricted diet, ammonia-reducing therapy, and L-carnitine supplementation, hyperammonemia was not observed, and liver enzyme levels returned to normal. *Conclusions*: Our results highlighted the clinical and biochemical heterogeneity of HHH syndrome and posed that HHH syndrome should be considered when individuals have hyperammonemia, elevated transaminase, and decreased prothrombin time.

## 1. Introduction

Hyperornithinemia–hyperammonemia–homocitrullinuria syndrome (HHH; OMIM 238970) is a rare urea cycle disorder [1]. HHH syndrome elucidates 1–3.8% of urea cycle disorders [2]. Ornithine carrier 1 deficiency causes HHH syndrome, characterized by failure of mitochondrial ornithine uptake, hyperammonemia, and accumulation of ornithine and lysine in the cytoplasm [1,3]. The carbamylation of increased cytoplasmic lysine forms homocitrulline (ε-amino-carbamoyl-lysine). Patients display protein intolerance, episodic vomiting, growth retardation, hepatomegaly, liver failure, coagulopathy, and neurological symptoms, including loss of consciousness, seizures, pyramidal signs, and cognitive impairment with or without behavioral problems [4]. Camacho summarized data from 122 patients with HHH syndrome and reported lethargy (62%), coma (33%), increased liver enzymes (52%), and coagulation disorders (49%) [5]. A protein-restricted diet did not affect long-term neurological impairments with pyramidal tract signs (75%), intellectual disability (66%), and myoclonic epilepsy (34%). However, clinical manifestations and age of disease onset can broadly vary among individuals, even in the same family [5]. The age of onset ranges from the neonatal period to adulthood. Those with neonatal onset seem normal for the first 24–48 h, followed by the onset of symptoms associated with hyperammonemia (poor feeding, vomiting, lethargy, low temperature, and rapid breathing). Those with later onset may display chronic neurocognitive deficits and/or unexplained seizures, spasticity, acute encephalopathy secondary to hyperammonemic crisis, or chronic liver dysfunction.

Hyperornithinemia–hyperammonemia–homocitrullinuria (HHH) syndrome can be diagnosed by mutations in the *SLC25A15* gene and marked by elevated ammonia, homocitrulline, and ornithine levels [6]. HHH syndrome differs from other defects due to high urinary homocitrulline and ornithine [7]. Hyperornithinemia is present in almost all patients; however, a small proportion does not exhibit hyperammonemia and homocitrullinuria. Therefore, genetic testing for *SLC25A15* variants accompanied by at least one of three metabolic traits, hyperornithinemia, hyperammonemia, and homocitrullinuria, is pivotal for a definite diagnosis of HHH syndrome [8]. Early diagnosis improves clinical outcomes [8]. Acute treatment of HHH syndrome is similar to other urea cycle disorders, whereas long-term treatment of HHH syndrome is similar to carbamoyl phosphate synthetase I and ornithine transcarbamylase deficiency [7]. Protein restriction, citrulline, arginine, supplementation of essential amino acids, and sodium benzoate/sodium phenylbutyrate are required.

The pathogenic variants in the *SLC25A15* gene, an autosomal recessive inheritance pattern, cause hyperornithinemia–hyperammonemia–homocitrullinuria (HHH) syndrome [2]. The *SLC25A15* gene is located on chromosome 13q14.11 and comprises seven exons encoding for isoform 1 of the ornithine carrier ORC1 with a length of 301 amino acids [9]. The substrate binding of the ORC1 includes Glu77, Arg179, and Glu180 residues, and the Asn74 and Asn78 are situated in the substrate binding pocket [10]. HHH syndrome has been more frequently reported in French-Canadian, Italian, Japanese [11], and Palestinian [12]. The major mutant alleles in the *SLC25A15* gene included p.Phe188del and p.Arg179*, identified in 45% of HHH patients [13]. The pathogenic variant, p.Arg179*, was identified in a 5-year-old Vietnamese boy who migrated to the USA [3,14]. In this study, we report the phenotype, genotype, treatment, and outcome of four Vietnamese patients diagnosed with HHH syndrome. To our knowledge, our study is the first report of HHH patients in Vietnam.

## 2. Materials and Methods

### 2.1. Individuals

The study involved retrospective and prospective analyses of four unrelated Vietnamese children diagnosed with HHH syndrome at the Center of Endocrinology, Metabolism, Genetic/Genomics and Molecular Therapy, Vietnam National Children’s Hospital. Clinical symptoms, including seizure, poor feeding, vomiting, lethargy, pyramidal signs, and intellectual disability, were followed.

### 2.2. Biochemical Parameters

Biochemical investigations included plasma ammonia, amino and acyl acid profiles, liver function, and coagulation.

Urinary organic acid analysis was conducted using gas chromatography–mass spectrometry (GC/MS). The urine specimen was soaked into two 2.5 cm × 2 cm blotting papers and subsequently dried naturally at room temperature for 4 h [15,16]. Then, the dried blotting papers were folded into a 2.5 mL syringe and placed in a 10 mL test tube. Distilled water (1.2 mL) was added to each tube and kept for 5 min. After centrifugation at 3000 rpm for 5 min, the supernatant was collected and transferred into a new 1.5 mL Eppendorf tube. We used two internal standards: margarate (MGA) and tetracosane (C24) (Sigma-Aldrich, Saint Louis, MO, USA). The 20 μg of each internal standard was added to the urine sample. Then, the distilled water was added to yield a final volume of 2 mL. One milliliter of 5% hydroxylamine hydrochloride (Sigma-Aldrich, Saint Louis, MO, USA) was added after adjusting the pH to 12–14 with 2N NaOH (Sigma-Aldrich, Saint Louis, MO, USA) and kept at room temperature for 60 min to oximize 2-ketoacids. Then, the resulting solution was acidified to pH 1.0 with 0.2 mL of 6N HCl (Sigma-Aldrich, Saint Louis, MO, USA), and 1 g of NaCl (Sigma-Aldrich, Saint Louis, MO, USA) was added. The urinary organic acids were extracted twice with 6 mL of ethyl acetate (Sigma-Aldrich, Saint Louis, MO, USA) and once with 6 mL of diethyl ether (Sigma-Aldrich, Saint Louis, MO, USA). After centrifugation, the organic layers were combined and dehydrated with 5 g of anhydrous sodium sulfate (Sigma-Aldrich, Saint Louis, MO, USA). Then, the supernatant was removed and dried with nitrogen at 60 °C. The extracted organic acids were derivatized by adding 100 μL of a mixture of N, O-bis(trimethylsilyl) trifluoroacetamide (BSTFA), and trimethylchlorosilane (TMCS) (10:1, *v*/*v*) (Sigma-Aldrich, Saint Louis, MO, USA). Afterward, they were kept at 80 °C for 30 min. Then, the derivatized solution was analyzed using a capillary GC/MS system (Shimadzu model QP 5000, Kyoto, Japan). The capillary column was a silica-coated DB-5 (30 m in length × 0.25 mm inside diameter) with a 1 μm film thickness of 5% phenyl methyl silicone (Shimadzu, Kyoto, Japan). The mass spectrum was formed by standard electron impact ionization scanning from the ion fragments with mass numbers from 50 *m*/*z* to 600 *m*/*z* at 0.4 s/rev. The initial temperature was 100 °C for 4 min, then increased to 290 °C at 4 °C/min and held constant for 10 min. The temperature of the injection port and transfer line was 280 °C. The flow rate of the helium gas was 1.5 mL/min, and the maximum velocity was 40.2 m/s. One microliter of the final derivatized solution was injected into the GC/MS for split-flow analysis. The urinary organic acids were analyzed using an automated data analysis system.

### 2.3. Genetic Testing and In Silico Analysis

Total genomic DNA samples were extracted from the whole blood samples using the QIAamp DNA Blood Mini Kit (Qiagen, Hilden, Germany). Genomic DNA samples were enriched for targeted regions employing a hybridization-based protocol and sequenced utilizing Illumina technology. The gene panel comprised 10 genes associated with urea cycle disorders. These genes were *ALDH18A1*, *ARG1*, *ASL*, *ASS1*, *CPS1*, *NAGS*, *OAT*, *OTC*, *SLC25A13*, and *SLC25A15*. Bioinformatics analyses and variant interpretation of the patients were performed at the Center for Gene-Protein Research, Hanoi Medical University. Reads were aligned to a reference sequence (GRCh37). Variants were identified and interpreted using relevant reference transcripts of NM_002860.3 for *ALDH18A1*, NM_000045.3 for *ARG1*, NM_000048.3 for *ASL*, NM_000050.4 for *ASS1*, NM_001875.4 for *CPS1*, NM_153006.2 for *NAGS*, NM_000274.3 for *OAT*, NM_000531.5 for *OTC*, NM_014251.2 for *SLC25A13*, and NM_014252.3 for *SLC25A15*.

The effect of variants was predicted using the Mutation Taster tool [17]. The pathogenicity of variants was determined using the ClinVar database. The frequency of variants was checked based on the information in the dbSNP154 database (https://www.ncbi.nlm.nih.gov/snp/, accessed on 2 May 2024) and gnomAD v4.1.0 database (https://gnomad.broadinstitute.org/, accessed on 2 May 2024).

### 2.4. Management

The patient management was based on the previous guidelines [7]. In managing acute hyperammonemia, the patients stopped all protein intake for 24–48 h and were given glucose infusion at 8–10 mg/kg/min, supplemental arginine 300–500 mg/kg/day, L-carnitine 100 mg/kg/day, and sodium benzoate 200–300 mg/kg/day and underwent extracorporeal detoxification. The long-term treatment for the patients involved a low protein diet, arginine of 300–500 mg/kg/day, L-carnitine of 100 mg/kg/day, and sodium benzoate of 200–300 mg/kg/day.

## 3. Results

### 3.1. Clinical Findings

One male and three females were involved in this study (Table 1). One neonatal onset case and three late-onset cases existed (Table 1). The age of onset was 7 days and 18–31 months in the neonatal and late-onset forms, respectively. Cases 2 and 4 were diagnosed immediately after onset; however, Cases 1 and 3 were diagnosed 15–17 months after onset. At the diagnosis, two cases exhibited poor feeding and episodic vomiting; three cases had lethargy, with four cases not displaying any pyramidal signs (Table 1). Hyperammonemia, elevated transaminases, and uraciluria were observed in three cases (Table 1). Biochemical analyses revealed hyperornithinemia and liver failure with elevated international normalized ratio (INR) but not homocitrulline in the urine in four cases. Four cases demonstrated biochemical heterogeneity (Table 1).

Case 1 is the second child of family 1, born by vaginal delivery. She had normal physical development. At 31 months of age, she had elevated transaminases and prolonged prothrombin time in the context of fever and fatigue episodes. Diagnosed with unknown liver failure, she was treated with arginine and vitamin K supplements for 14 months. At 46 months of age, she displayed fatigue and lethargy and was admitted to our department. After analyzing for plasma ammonia and amino and acyl acid analyses, she indicated hyperammonemia (98.0 μmol/L) and hyperornithinemia (258.8 μmol/L) with elevated transaminases and uraciluria. The family history illustrated normal conditions.

Case 2 is the second child of family 2, also born by vaginal delivery. After the birth, she had symptoms of poor feeding, vomiting, jaundice, and lethargy and was admitted to our department. Biochemical investigations yielded hyperammonemia (192.0 μmol/L), hyperornithinemia (256.5 μmol/L), and normal levels of transaminases. The urine analysis revealed normal uracil, orotic acid, and homocitrulline levels. Her brother exhibited normal development.

Born by vaginal delivery, Case 3 is the second child of family 3. At 31 months of age, he exhibited fever, cough, fatigue, irritability, lethargy, hypertransaminase, and prolonged prothrombin time. Hence, he was diagnosed with liver failure and treated with glucose infusion, arginine, and vitamin K. At 48 months of age, he exhibited fatigue and lethargy and was admitted to our department. Biochemical investigations indicated slight hyperammonemia (76.8 μmol/L), hyperornithinemia (329.2 μmol/L), and high transaminases (Alanine transaminase 1055 UI/L and aspartate transaminase 1033 UI/L). Increased uracil was detected in the urine sample.

Case 4 is the first child of family 4, born by vaginal delivery. She displayed normal physical development. At 18 months of age, she had a seizure (under 10 s) and vomiting and was admitted to our department. Biochemical investigations revealed hypertransaminases (Alanine transaminase 69 UI/L and aspartate transaminase 58 UI/L), hyperornithinemia (193.7 μmol/L), and prolonged prothrombin time, but normal levels of ammonia (49.5 μmol/L).

### 3.2. Molecular Findings

Through molecular analyses of the four patients, we identified three variants in the *SLC25A15* gene classified as pathogenic variants according to ClinVar (Table 2). The variant c.535C>T (p.Arg179*) was a common variant, which was present in all four patients. Cases 1 and 2 were homozygous for the c.535C>T (p.Arg179*) variant. Case 3 harbored compound heterozygosity for c.408delC (p.Met137Cysfs*10) and c.535C>T (p.Arg179*). Case 4 had compound heterozygous variants of c.535C>T (p.Arg179*) and c.562_564delTTC (p.Phe188del).

The variant c.408delC (p.Met137Cysfs*10) has not been reported in the literature on HHH patients. The variant was reported by Invitae and Baylor Genetics in the ClinVar database (ID 85164) as a pathogenic or likely pathogenic variant. The variant c.408delC was observed in an East Asian at the heterozygous state (https://gnomad.broadinstitute.org/variant/13-40805209-AC-A?dataset=gnomad_r4, accessed on 2 May 2024). The variant was predicted as a deleterious variant in the Mutation Taster tool.

### 3.3. Treatment and Outcome

Cases 1 and 3 were misdiagnosed with unknown liver failure and treated with vitamin K1 (phytok 5 mg/day) and arginine supplement. However, their liver functions only improved once they were diagnosed accurately. After an accurate diagnosis, four cases were subjected to a protein-restricted diet (1–1.5 g/kg/day), L-carnitine (100 mg/kg/day), arginine (300–500 mg/kg/day), and sodium benzoate (100–250 mg/kg/day) (Table 3). All cases responded well to the treatment, depicting no acute hyperammonemia. Cases 1, 2, 3, and 4 were discharged after 10, 10, 7, and 5 days of treatment, respectively. After 3 days of treatment, Cases 1 and 2 rapidly returned to normal levels of transaminase and coagulation. Nevertheless, in Case 4, transaminases decreased gradually to normal levels after 2 months of treatment. Meanwhile, in Case 3, liver enzyme and blood clotting index slowly improved and returned to normal after 2 years of treatment. In the last visit, all cases depicted normal levels of ammonia, normal brain magnetic resonance imaging, and normal physical development. Cases 1, 2, and 3 had slightly increased levels of transaminases and international normalized ratio (INR), with Case 4 demonstrating normal levels. Case 3 displayed attention deficit hyperactivity disorder (Table 3). At the age of five, Case 3 was diagnosed with mild deficit hyperactivity disorder and received psychotherapy intervention at home. Case 3 is six years old now and has improved and increased focus on learning.

## 4. Discussion

Hyperornithinemia–hyperammonemia–homocitrullinuria (HHH) syndrome is a sporadic disorder of the urea cycle in Vietnam. Until now, only four cases have been diagnosed in our center. The diagnosis was based on clinical, biochemical, and molecular analyses.

In our study, we observed diverse onset ages and delayed diagnoses. The age of onset ranged from neonatal to toddler. Case 2 was presented at 10 days old, which aligned with the findings of Camacho and colleagues [5], who posed that the neonatal onset rate was about 8% in people with HHH syndrome. This onset rate usually appears 24–48 h after breastfeeding with acute symptoms [5]. Martinelli and colleagues (2015) reported symptoms in the neonatal period for 2% of patients, 24% from 1 month to under 1 year old, and 44% from 1–12 years old [2]. Even though up to 1/3 of children have symptoms of neonatal onset, diagnosis is often delayed, with an average diagnostic delay of 6.3 ± 10.1 years (range 0–37 years) [2]. In our study, Cases 1 and 3 were misdiagnoses of unknown liver failure, with the accurate diagnosis delayed 15 months and 17 months, respectively. The clinical symptoms of children with HHH syndrome are diverse and nonspecific [2,6,12]. For example, due to hyperammonemia, HHH patients exhibited acute neurological symptoms, including seizures, poor appetite, vomiting, and lethargy. Therefore, these HHH patients can easily be misdiagnosed with encephalitis, epilepsy, cerebral hemorrhage, or poisoning. Additionally, hepatosplenomegaly can not immediately suggest a metabolic disorder. Other symptoms encompass acute encephalopathy, chronic liver disease, or cognitive impairment/learning disability/seizures.

Four cases also depicted biochemical heterogeneity. Three of them were presented with hyperammonemia. The median blood ammonia concentration usually ranged from 100 to 300 µmol/L, with newborns having higher average blood ammonia concentrations than older children and adults [5]. Wild et al. reported that a premature infant diagnosed with HHH syndrome had a blood ammonia concentration of 1300 µmol/L when they received intravenous nutrition and 623 µmol/L after stopping intravenous nutrition [1]. In our study, Case 3, which was early onset at 7 days, had the highest ammonia level (192 µmol/L). Case 4 displayed a normal level of ammonia but positive oroticaciduria at the diagnosis, contrasting with the other patients. The normal level of ammonia and elevated levels of transaminases caused Case 4 to be mistaken for liver failure of unknown cause or autoimmune hepatitis. Therefore, we recommend that patients with unexplained liver failure be repeatedly tested for ammonia primary when a change in consciousness and inborn errors of metabolism screening exist, such as MS/MS and plasma amino acids.

In our study, four cases had hyperornithinemia, which is observed in HHH patients [2,18]. Martinelli and colleagues reported that blood ornithine concentrations of patients with HHH syndrome increased from 216 to 1915 µmol/L (Normal: 30–110 µmol/L) [2]. Despite treatment with medication and a protein-restricted diet, blood ornithine levels remained elevated, and only a few patients were reported to have normal levels upon long-term follow-up [2]. Thus, the blood ornithine index was reliable in newborn screening to help with early diagnosis of HHH syndrome. No homocitrulline was detected in the urine of the four cases. Homocitrullineuria is a characteristic sign of the disease; however, patients who may have no or only marginal homocitrulline excretion in the urine are present [12,19]. Especially in neonates, homocitrulline may be obscured by plasma amino acid profile and abnormal aminoaciduria in liver dysfunction [5]. Such factors may have impacted the detection of urinary homocitrulline in our four cases.

**Figure 1:**
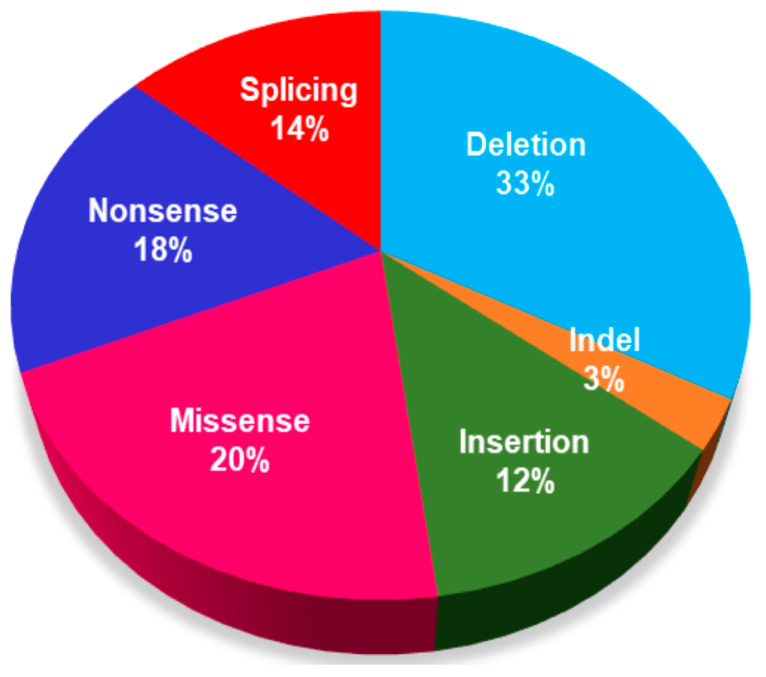
Distribution of pathogenic or likely pathogenic variants in the *SLC25A15* in the ClinVar database. All 73 pathogenic or likely pathogenic variants were listed in ClinVar database (accessed on 23 July 2024).

Our study involved six of eight alleles of c.535C>T (p.Arg179*), suggesting that it was a “hot spot” variant in Vietnamese patients with HHH syndrome. The c.535C>T (p.Arg179*) variant was one of the most common variants observed in various HHH patients, including Japanese, Italian, Senegal, Morocco, Han Chinese, Korean, and Thailand [2]. Therefore, the c.535C>T (p.Arg179*) variant might have a broader carrier distribution in Vietnamese and could be used in population screening programs. Another common variant, c.562_564delTTC (p.Phe188del), which was reported in French-Canadian [11,18,20], Italian [21], Korean [22], and Pakistan [23], was identified in one of our patients. These two variants are located in exon 4 of the *SLC25A15* gene. Thirteen pathogenic or likely pathogenic variants are reported in exon 4 in the ClinVar database. The third variant, c.408delC (p.Met137Cysfs*10), is situated in exon 3. This variant causes a frameshift; methionine at the position of amino acid 137 changes to cysteine and early termination after mutation of 10 amino acids. The identified variants in our study are nonsense or deletion variants aligning with the ClinVar database (accessed on 23 July 2024). Figure 1 depicts that the nonsense and deletion variants contribute 18% and 33% of the total pathogenic or likely pathogenic variants in the *SLC25A15* gene, respectively.

Both Cases 1 and 2 harbored the same nonsense variant c.535C>T (p.Arg179*) at the homozygous state. However, they posed clinical heterogeneity. Case 2 depicted neonatal onset at 7 days, whereas Case 1 demonstrated the late-onset form at 31 months. Table 4 displays thirteen previously reported patients harboring c.535C>T/c.535C>T with the details of clinical and biochemical characteristics selected for comparison. Overall, 17 patients with c.535C>T/c.535C>T variant exhibited diverse phenotypes with the typical characteristics of lethargy, hyperornithinemia, hyperammonemia, and elevated homocitruline (Table 4). Seven patients were diagnosed at very late onset (>10 years), whereas three presented symptoms during the neonatal period. Nevertheless, they depicted different phenotypes in coma, coagulopathy, seizure, pyramidal signs, and levels of ornithine and ammonia [23]. Table 4 illustrates that our patient (P17) had normal ammonia levels, aligning with five Turkish patients. However, the five Turkish patients did not depict any medical history of hyperammonia. The probands P8 and P11 were referred for gait disturbances [6], and genetic analysis revealed that they harbored homozygotes of c.535C>T (p.Arg179*). Then, their siblings were screened for the pathogenic variant. Results demonstrated that the siblings also carried the pathogenic variant and presented with elevated ornithine and homocitruline. However, they did not exhibit any symptoms of HHH syndrome. No correlation between the genotype and phenotype in HHH patients seemed to exist [12,20,23]. To our knowledge, no published data to date of patients harboring compound heterozygous variants *SLC25A15*: c.535C>T/c.408delC or c.535C>T/c.562_564delTTC are present.

Before obtaining accurate diagnoses, the liver functions of Cases 1 and 3, managed with vitamin K1 and arginine supplement, had not improved over 15 and 17 months. After a protein-restricted diet, L-carnitine, arginine, and sodium benzoate, liver functions in all cases became normal. For Cases 1 and 2, liver enzyme levels returned to normal after 3 days of treatment. Nonetheless, it took 2 years and 2 months for Cases 3 and 4, respectively. All cases had not occurred with the acute crisis of hyperammonemia for 2–5 years because they were prevented by enough energy support (glucose infusion) in the case of stresses (fever, vomiting, vaccination, etc.), aligning with other studies. All of our patients exhibited normal physical development. Therefore, early, accurate diagnosis of HHH syndrome is necessary more than ever, especially in newborn screening. Diet therapy, controlling plasma ammonia levels, and preventing acute crises from stresses provide better outcomes.

No brain abnormalities were detected in the brain MRI of our four cases. Neuroradiological abnormalities usually occur after 20 years of age [28]. In our study, brain MRI was performed at 4 to 9 years of age. Hence, neurological abnormalities have not been illustrated in the MRI findings. Additionally, after age 15, HHH patients developed spastic paraparesis, which was not related to dietary intake and sodium benzoate treatment [12]. Therefore, HHH patients should be carefully monitored by clinical examination and spinal and brain MRI for the long term. We suggest that an MRI be performed annually after 15 years of age.

Our study’s limitations include a small sample size and lack of functional testing to demonstrate the effect of pathogenic variants on protein functions in vitro or in vivo. Further studies would be needed to reach conclusive conclusions.

## 5. Conclusions

Hyperornithinemia–hyperammonemia–homocitrullinuria (HHH) syndrome is a clinical and biochemical heterogeneity disorder. The clinical spectrum is diverse, and biochemical changes are nonspecific. HHH syndrome should be considered when evaluating individuals with unexplained hyperammonemia or persistently elevated liver enzymes and decreased prothrombin ratio, especially in newborn screening. The hot spot at residue 179 of SLC25A15 in Vietnamese cases with HHH syndrome may be used in the screening of individuals suspected of HHH syndrome.

## Figures and Tables

**Table 1:** Clinical features and biochemical profile of the four cases.

	Case 1	Case 2	Case 3	Case 4
Sexuality	Female	Female	Male	Female
Age of onset	31 months	7 days	31 months	18 months
	Elevated transaminases and prolonged prothrombic time	Poor feeding, vomiting, jaundice, lethargy	Fever, lethargy, irritability, elevated transaminases, and prolonged prothrombic time	Vomiting and seizure
Age of diagnosis	46 months	10 days	48 months	19 months
Poor feeding	+	+	−	−
Episodic vomiting	+	+	−	−
Seizure	−	−	−	−
Lethargy	+	+	+	−
Hepatosplenomegaly	−	−	+	−
History of fibrile seizure	−	−	−	+
**Biochemical profile at the diagnosis**				
Hyperammonemia (μmol/L)	+ 98.0	+ 192.0	+ 76.8	−49.5
Hyperornithinemia (μmol/L)	+ 258.8	+ 256.5	+ 329.2	+ 193.7
Elevated transaminases	+	−	+	+
✓ Alanine transaminase (UI/L)	118	30	1055	69
✓ Aspartate transaminase (UI/L)	56	19	1033	58
Prolonged Prothrombin time (%)	32	46	48	46
International normalized ratio (INR)	1.67	1.52	1.55	1.74
Uraciluria	+	−	+	+
Oroticaciduria	−	−	−	+
Homocitrullinuria	−	−	−	−

(+), present; (−) not present.

**Table 2:** Molecular analyses of the four cases.

Patient	Gene	Variant	dbSNP152	ClinVar
Case 1	*SLC25A15*	c.535C>T (p.Arg179*)/c.535C>T (p.Arg179*)	rs104894429	5994 (Pathogenic)
Case 2	*SLC25A15*	c.535C>T (p.Arg179*)/c.535C>T (p.Arg179*)	rs104894429	5994 (Pathogenic)
Case 3	*SLC25A15*	c.535C>T (p.Arg179*)/	rs104894429	5994 (Pathogenic)
		c.408delC (p.Met137Cysfs*10)	rs780201405	851,641 (Pathogenic)
Case 4	*SLC25A15*	c.535C>T (p.Arg179*)/	rs104894429	5994 (Pathogenic)
		c.562_564delTTC (p.Phe188del)	rs202247803	5992 (Pathogenic)

**Table 3:** Treatment and outcome of the four HHH cases.

	Case 1	Case 2	Case 3	Case 4
**Treatment before accurate diagnosis**				
Age of treatment	31 months	None	31 months	None
Vitamin K1				
Arginine supplement (mg/kg/day)	300–500	None	300–500	None
**Treatment after accurate diagnosis**				
Age of treatment	46 months	10 days	48 months	19 months
**Low protein diet (g/kg/day)**				
L-carnitine supplement (mg/kg/day)	100	100	100	100
Arginine supplement (mg/kg/day)	300–500	300–500	300–500	300–500
Sodium benzoate supplement at the Acute episodes (mg/kg/day)	100–250	100–250	100–250	100–250
**Outcomes**				
Treatment time to achieve normal transaminase and coagulation	3 days	3 days	2 years	2 months
Current age	9 years	4 years	6 years	4 years
Physical development ✓ Height ✓ Weight	Normal −0.6 SD −1.4 SD	Normal −1.8 SD −1.8 SD	Normal −0.4 SD −0.4 SD	Normal −1.5 SD −0.6 SD
Attention deficit hyperactivity disorder	None	None	Yes	None
Brain magnetic resonance imaging	Normal	Normal	Normal	Normal
Relapse	None	None	None	None
**Biochemical profile at the last visit**				
Plasma ammonia level (µmol/L)Transaminases	29.4	28.2	21.0	10.2
✓ Alanine transaminase (UI/L) ✓ Aspartate transaminase (UI/L)	34 47	50 44	47 55	39.3 32.4
Prothrombin time (%)International normalized ratio (INR)	73 1.25	75 1.22	61 1.43	81 1.15

**Table 4:** Clinical characteristics of patients with homozygote c.535C>T (p.Arg179*).

Cases	Sex	Ethnicity	Onset Age	Diagnosis Age	Lethargy	Coma	Elevated Transaminases	Coagulopathy	Intellectual Disability	Seizures, Myoclonic	Pyramidal Signs	Ornithine (μmol/L)	Ammonia (μmol/L)	Elevated Homocitrulline	Ref.
P1	m	Japanese	3.0 y	10.0 y	+	−	−	−	Severe	−	+	↑ 419	↑ 204	+	[24]
P2	m	Japanese	11.0 y	41.0 y	+	+	−	−	Mild	−	+	↑ 586	↑ 98	+	[25]
P3	f	Japanese	NR	52.0 y	−	−	−	−	−	+	+	↑	↑ 242	+	[26]
P4	m	Italian	12.0 y	26.0 y	+	−	−	+	Mild	+	+	NR	↑	NR	[27]
P5	f	Senegal	Birth	Birth	+	+	−	+	Mild	+	+	↑ 509	↑ 700	+	[23]
P6	f	Senegal	Birth	Birth	+	−	−	−	Mild	−	+	↑ 290	↑ 100	+	[23]
P7	m	Morocco	1.7 y	1.7 y	+	−	+	+	−	−	−	↑ 493	↑ 96	+	[23]
P8	m	Turkish	16.0 y	16.0 y	−	−	−	−	−	−	+	↑ 367	44	+	[6]
P9	f	Turkish	10.4 y	10.4 y	−	−	−	−	−	−	-	↑ 468	37	+	[6]
P10	m	Turkish	2.3 y	2.3 y	−	−	−	−	−	−	-	↑ 305	50	+	[6]
P11	m	Turkish	13.1 y	14.0 y	−	−	−	−	−	−	+	↑ 446	40	+	[6]
P12	f	Turkish	17.5 y	17.5 y	−	−	−	−	−	−	-	↑ 248	27	+	[6]
P13	f	Vietnamese	1.0 y	5.0 y	+	+	+	+	Moderate	+	+	↑ 1439	↑ 353	−	[3]
P14	f	Vietnamese	2.6 y	3.8 y	+	−	+	+	−	−	−	↑ 259	↑ 98	−	This study
P15	f	Vietnamese	7 d	10 d	+	−	−	+	−	−	−	↑ 256	↑ 192	−	This study
Summary					9/15 (60%)	3/15 (20%)	3/15 (20%)	6/15 (40%)	6/15 (40%)	4/15 (27%)	9/15 (60%)	14/15 (93%)	10/15 (67%)	11/15 (73%)	

m, male; f, female; y, year; NR, not recorded; +, present; −, not present; ↑, elevated.

## Data Availability

The raw data supporting the conclusions of this article will be made available by the authors on request.
